# Applied biodiversity science in China in the global context

**DOI:** 10.1093/nsr/nwab059

**Published:** 2021-04-09

**Authors:** Thomas M Brooks, Xinsheng Zhang

**Affiliations:** International Union for Conservation of Nature, Switzerland; International Union for Conservation of Nature, China

In 2021, the city of Kunming in Yunnan Province, China, will host the fifteenth meeting of Conference of Parties of the Convention on Biological Diversity. The meeting is expected to be the pivotal moment at which the world's governments agree on a new ‘Post-2020 Global Biodiversity Framework’ (https://www.cbd.int/conferences/post2020). The framework is anticipated to be structured around a small number of outcome goals for 2050—a compelling case has been made that these should include explicit goals for ecosystems, species, genetic diversity and the contributions which nature makes to people [1]. These would support both the 2050 Vision of the Convention on Biological Diversity and the other seven biodiversity-related conventions (https://www.cbd.int/brc/), as essential components of the UN Sustainable Development Goals (https://sustainabledevelopment.un.org/sdgs). They would in turn be supported by perhaps 20 action targets for 2030, the implementation of which would be necessary and sufficient to deliver the goals. Crucially, this structure would incentivize the establishment of ‘science-based targets’ for explicit contributions towards the goals from all actors [2], allowing ‘mainstreaming’ of biodiversity across all of society.

In the context of this essential role, which China will serve over 2021 and beyond in advancing the global commitment to safeguard life on Earth, Mi *et al.*’s sweeping overview of ‘the global significance of biodiversity science in China’ [3] is highly timely. They review the output led by Chinese institutions and published in interdisciplinary international journals over the last two decades (nearly 200 papers in total), across three broad categories: inventory and monitoring; processes and mechanisms; and threats and responses. Based on this, they propose priorities for future research, mechanisms for strengthening translation of science into biodiversity conservation practice, prospects for harnessing new technology and pathways for advancing international collaboration. Here, we build from the foundation established by Mi *et al.*’s impressive synthesis to offer suggestions as to five specific opportunities for strengthening the science–policy interface for biodiversity in China in the global context.

## TIGHTENING INTERLINKAGE BETWEEN NATIONAL AND GLOBAL RED LISTS

Mi *et al.* provide powerful documentation of the excellent progress made in recent decades in documenting the extinction risk facing China's species [3]. Placing this progress into the context of global assessment of extinction risk is necessary to document additive national contributions towards global goals in the way essential for delivery of the Post-2020 Global Biodiversity Framework [4]. Consideration of the national Red List data presented in Mi *et al.*’s Table 1, alongside the equivalent global data from the IUCN Red List of Threatened Species [5] (https://www.iucnredlist.org/), reveals considerable opportunity to strengthen such national–global linkage (Table 1).

**Table 1.** Comparison between numbers of species, endemic species, and threatened species as documented by the Table 1 from Mi *et al.* [3] and by the IUCN Red List [5].

	Species	Species	Endemics	Endemics	Threatened	Threatened
Source	[3]	[5]	[3]	[5]	[3]	[5]
Bryophytes^1^	3021	10	524	2	186	9
Ferns^2^	2129	52	842	23	182	20
Gymnosperms^3^	237	181	88	119	148	65
Angiosperms^4^	28 996	3169	14 693	1089	3363	575
Amphibians	408	455	272	229	176	141
Reptiles	461	334	142	96	137	51
Birds	1372	1327	77	68	146	102
Mammals	673	605	150	94	178	88

^1^Encompasses Bryophyta, Marchantiophyta and Anthoceratophyta. ^2^Polypodiopsida. ^3^Encompasses Pinopsida, Cycadopsida, Gnetopsida and Ginkoopsida. ^4^Encompasses Magnoliopsida and Liliopsida.

The explanation for the much greater numbers of bryophyte, fern and angiosperm species assessed in the national Red List is simple—assessments of most of these species have not yet been included in the global Red List. Given the large numbers of Chinese endemic species within these plant groups, a priority action could be to use the SISconnect tool to upload assessments of these endemics directly to the IUCN Red List. This is straightforward for national endemics where national assessments have been undertaken using the ‘Guidelines for Application of IUCN Red List Criteria at Regional and National Levels’ [6], not least because the IUCN Red List includes assessments in any language, including Chinese. Interlinkage for the other five species groups, which have been comprehensively assessed at both national and global levels, is more complex because mismatches are likely explained by a range of different factors [7]—for example, some species may genuinely be nationally but not globally threatened, while in other cases the underlying taxonomy used may differ. Nevertheless, consistent assessment of national endemics, at least, would be greatly beneficial in allowing China to document progress towards global biodiversity goals over the coming years.

## ASSESSING RISK OF ECOSYSTEM COLLAPSE

One of the advances in biodiversity science in China described by Mi *et al.* [3] is the meticulous development of vegetation maps for the country. The likelihood that the Post-2020 Global Biodiversity Framework will incorporate an ecosystems goal presents an opportunity to build from these vegetation maps towards internationally comparable assessment of risk of ecosystem collapse across China. Until recently, the limiting factor facing such an effort has been the lack of a scaleable, globally comprehensive ecosystem typology. Such a typology was published at the end of 2020 [8] (https://global-ecosystems.org/), structured to allow national classifications such as vegetation maps of China to nest within it. An important research priority remains the development of equivalent classification for non-vegetated ecosystems of China, especially for marine and freshwater biomes—both of which are highlighted as research priorities by Mi *et al.* With a national-within-global ecosystem typology in place, it would then become possible to apply standard categories and criteria [9,10] to assess risk of collapse of China's ecosystems (https://iucnrle.org/), in support of setting targets and monitoring indicators towards the ecosystem goal likely to be established under the Post-2020 Global Biodiversity Framework.

## ADDRESSING DATA DEFICIENCY AND TAKING CITIZEN SCIENCE TO SCALE

**Figure 1.**
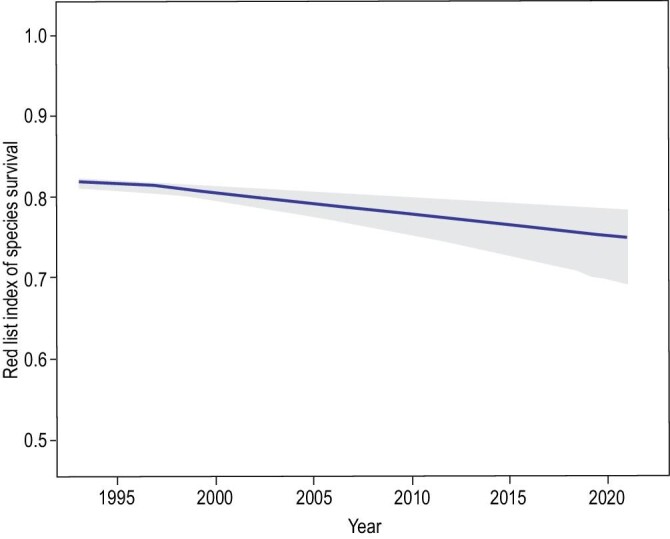
Current Red List Index for China, the official indicator of progress towards SDG 15.5 (source: https://www.ibat-alliance.org/ country_profiles/CHN).

Another theme emerging as an important priority from Mi *et al.*’s review [3] is the prospect of targeted efforts to reduce data deficiency for China's biodiversity. Of the 9942 Chinese species currently assessed on the IUCN Red List [5], no less than 1315 are categorized as ‘Data Deficient’, that is, lacking sufficient data to determine their extinction risk. This has important policy implications—for example, it is this data deficiency which introduces uncertainty around the Red List Index, which is generated by measuring changes in extinction risk between Red List assessments, excluding non-genuine changes resulting from changing knowledge [11,12]. This is the indicator used to track progress towards Sustainable Development Goal 15.5 (https://unstats.un.org/sdgs/iaeg-sdgs/), and is proposed as a headline indicator for the Post-2020 Global Biodiversity Framework. The uncertainty around the current Red List Index for China is > 10% (Fig. 1).

**Figure 2.**
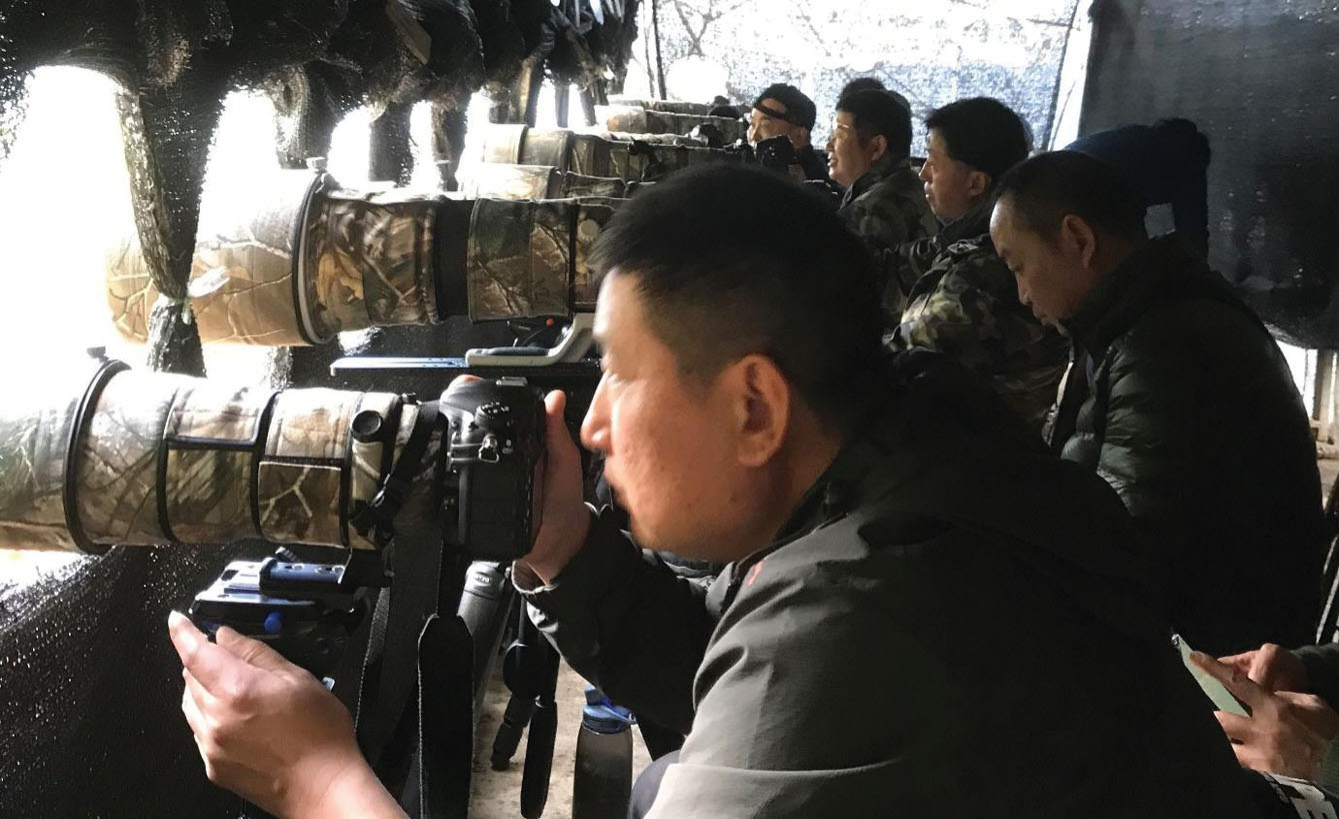
The positive prospects for citizen science in China illustrated by bird photographers in Baihualing, Gaoligongshan, Yunnan Province, in December 2018 (photograph: T.M. Brooks).

A number of approaches could be deployed to reduce this data deficiency. One strategy which has productively been deployed elsewhere is to target graduate student field research onto individual Data Deficient species. Given the explosive growth of biodiversity science in China over the last two decades, illustrated so well in Mi *et al.*’s Fig. 1, the country certainly has the volume of early career researchers needed to address such a challenge. A complementary approach, also noted by Mi *et al.*, could be to harness citizen science more effectively. While citizen scientists in China have to date uploaded 63 500 checklists (3218 observers) to eBird (https://ebird.org/ region/CN) and 137 797 observations (4947 observers) to iNaturalist (https://www.inaturalist.org/places/china), the equivalent statistics for, say, India, are 1 200 000 checklists (22 400 observers) and 512 472 observations (14 478 observers), respectively. Collaboration between Chinese and international institutions to develop national-customized interfaces to such platforms could yield huge dividends in increasing the volume of citizen science data. The popularity of natural history photography in China (Fig. 2) also means that mechanisms for verification, geolocation and time-stamping of citizen science observations are easily available.

## TARGETING PROTECTED AREA COVERAGE OF KEY BIODIVERSITY AREAS AS ECOLOGICAL CONSERVATION REDLINING

One of Mi *et al.*’s most important recommendations concerns translation of scientific advances into biodiversity conservation actions, especially in the context of the country's ecological conservation redline policy [13]. A number of priority activities stand out here as necessary to ensure that China's designation of protected areas and other effective area-based conservation measures are, indeed, effectively safeguarding biodiversity.

First, robust data on Chinese sites contributing significantly to the global persistence of biodiversity are a crucial input into effective ecological conservation redlining. The recent establishment of ‘A Global Standard for the Identification of Key Biodiversity Areas’ [14] provides the framework for generation of such data. Already, 667 key biodiversity areas have been identified in China [15], although there is a taxonomic focus on important sites for birds, comprising 512 of these sites. Establishment of a China national coordination group (http://www. keybiodiversityareas.org/working-with-kbas/programme/ national-coordination-groups) to advance comprehensive application of the standard across other taxonomic groups and ecosystems would represent a great advance.

Second, it is also essential to maintain up-to-date data on protected areas and other effective area-based conservation measures, which are compiled from national data sources through Protected Planet [16], under the mandate of the UN List of Protected Areas. While protected areas are sites with explicit management objectives for biodiversity conservation [17], other effective area-based conservation measures are managed for other objectives but nevertheless deliver biodiversity outcomes [18]. Currently, 122 Chinese protected areas are documented (https://www.protectedplanet.net/country/CHN), but as yet no other effective area-based conservation measures have been documented in the country.

Third, as these crucial underlying datasets are progressively refined, the robustness of emergent products in informing policy and practice will improve accordingly. Thus, for example, combining the datasets yields the annual indicator of protected area coverage of key biodiversity areas [19], which is used to track progress towards Sustainable Development Goals 14.5 (for marine sites), 15.1 (for terrestrial and freshwater sites) and 15.4 (for mountains) (https://unstats.un.org/sdgs/iaeg-sdgs/). Moreover, information about the biodiversity for a given protected area is important and essential in focusing management activities—to date, just 19 Chinese protected areas have documented management effectiveness in Protected Planet, while 21 are either listed or candidate sites for the IUCN Green List of Protected and Conserved Areas (https:// iucngreenlist.org/).

## DOCUMENTING AND ADDRESSING BIODIVERSITY IMPACTS EMBODIED IN INTERNATIONAL TRADE AND INVESTMENT

The final recommendation emerging from Mi *et al.*’s review [3] concerns strengthening international collaborations. While this is relevant to all of the themes discussed above, it is particularly important in the context of addressing biodiversity impacts embodied in trade and investment. Mi *et al.* specifically discuss the importance of engagement with the Belt and Road Initiative, to safeguard biodiversity along its projected routes—for example, its railways are anticipated to pass within 1 km of 440 key biodiversity areas, and its roads within 1 km of 937 key biodiversity areas [20]. The issue of embodied impacts also concerns effects on biodiversity imported and exported through international trade, the assessment of which is increasingly being enabled by application of techniques from environmentally extended input-output analysis [21]. Hand-in-hand with the development and application of methods to allow documentation of such impacts embodied in international investment and trade, it then becomes possible to address them, for example through the establishment of standards and safeguards for financial institutions, such as those used in Performance Standard 6 of the International Finance Corporation (http://www.ifc.org/ps6) and the Equator Principles Banks (https://equator-principles.com/).

## CONCLUSION

Over recent decades, China has embodied both great success and great tragedy for biodiversity nationally and globally. On the one hand, efforts in the country have been rightly celebrated, in, for example, preventing the extinction of the Asian Crested Ibis (*Nipponia nippon*), Père David's Deer (*Elaphurus davidianus*), Przewalski's Horse (*Equus ferus*), and Hainan Gibbon (*Nomascus hainanus*) [22]. On the other, seven species are documented to have been driven globally extinct in China over recent decades—three of them from within 100 km of Kunming City: Yunnan Lake Newt (*Cynops wolterstorffi*) and the fishes *Anabarilius macrolepis* and *Cyprinus yilongensis*—and a further 26 are assessed as ‘Possibly Extinct’ [5].

The enormous upswing of biodiversity science in China over the last two decades documented by Mi *et al.* [3] is therefore highly encouraging. The upcoming Conference of the Parties of the Convention on Biological Diversity in Kunming in 2021 provides a splendid platform both to avoid further losses and to build from and accelerate such advances within the global context. This would allow China to jumpstart implementation of what will become its commitments to the new Post-2020 Global Biodiversity Framework, and then realize Mi *et al.*’s projection of becoming a global leader not only in biodiversity research but also in the policy and practice of its conservation in the near future.

***Conflict of interest statement.*** None declared.
